# Processing medical data: a systematic review

**DOI:** 10.1186/0778-7367-71-27

**Published:** 2013-10-09

**Authors:** Kasaw Adane, Dagnachew Muluye, Molla Abebe

**Affiliations:** 1 College of Medicine and Health Sciences, School of Biomedical and Laboratory Sciences, Unit of Laboratory Management and Quality Assurance, University of Gondar, Gondar, Ethiopia; 2 Department of Medical Microbiology, College of Medicine and Health Sciences, School of Biomedical and Laboratory Sciences, University of Gondar, Gondar, Ethiopia; 3 Department of Clinical Chemistry, College of Medicine and Health Sciences, School of Biomedical and Laboratory Sciences, University of Gondar, Gondar, Ethiopia

**Keywords:** Medical data, Documentation, Decision making, Electronic health record, Health service quality

## Abstract

**Background:**

Medical data recording is one of the basic clinical tools. Electronic Health Record (EHR) is important for data processing, communication, efficiency and effectiveness of patients’ information access, confidentiality, ethical and/or legal issues. Clinical record promote and support communication among service providers and hence upscale quality of healthcare. Qualities of records are reflections of the quality of care patients offered.

**Methods:**

Qualitative analysis was undertaken for this systematic review. We reviewed 40 materials Published from 1999 to 2013. We searched these materials from databases including ovidMEDLINE and ovidEMBASE. Two reviewers independently screened materials on medical data recording, documentation and information processing and communication. Finally, all selected references were summarized, reconciled and compiled as one compatible document.

**Result:**

Patients were dying and/or getting much suffering as the result of poor quality medical records. Electronic health record minimizes errors, saves unnecessary time, and money wasted on processing medical data.

**Conclusion:**

Many countries have been complaining for incompleteness, inappropriateness and illegibility of records. Therefore creating awareness on the magnitude of the problem has paramount importance. Hence available correct patient information has lots of potential in reducing errors and support roles.

## Background

Quality System Essentials (QSEs) are necessary to support any healthcare service’s workflow. They help to effectively manage and smoothly run work operations. If QSEs are not well implemented, work will experience problems [1]. Recording and documentation is one of the universal sets of policies, processes, and procedures applicable to all organizations [2]. It depends on rules, regulations, standards, and guidelines which improved using training, education and is a requirement for accreditation [3]. Documented quality management programs must be used to ensure quality service provision [4].

Clinical services information management system ensures the provision of appropriate and timely information to all stakeholders [5]. Clinical record and documentation is one of the most basic professional responsibilities even if it is often seen poorly practiced. Healthcare providers communicate patient information through clinical recording and communication systems [6]. Record keeping and information management are the requirements for the provision of quality patient service. They are the factors for the development of electronic records. Complete, integrated, and legible electronic records are important to allow the information access from multiple sites and generate risk alerts [7].

In light of the above mentioned reasons, this paper provides important points on recording, documentation, information processing, and communication of patient information related to quality healthcare provision.

## Methods

**Figure 1:**
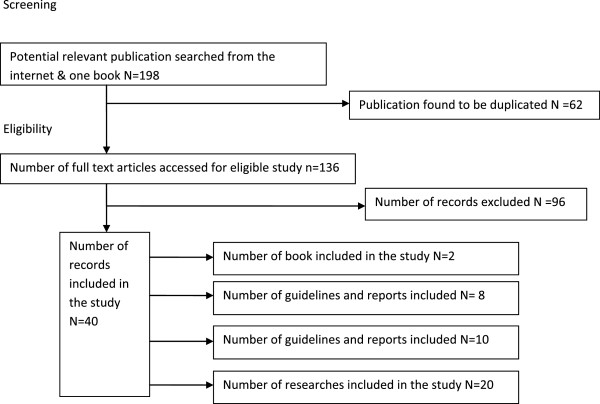
Flow diagram showing reviewed documents.

Qualitative method was used to evaluate the significance, actual practice, and problems related to processing medical data. Authors reviewed different guidelines, standards, journals, policies, reports (WHO recommendations), 2 books and related documents. Accessible materials were browsed from Internet sources which were published from 1999 to 2013. The following sites and search engines were used: HINARI [http://www.who.int/hinari/], Medline (Pubmed and OVID), Google scholar, and Science Direct. The selection process is as illustrated in Figure 1.

### Medical record and documentation

Revised health service processes and procedures can be implemented to improve providers’ performance [8]. This provides essential information for employees [9,10]. Therefore, well-trained health professionals, high quality facility, equipment, and good record keeping systems are important for health institutions like hospitals. Patient records and registers must be kept confidential and protected. It saves time as well as increases the chance of getting the required data [11].

Reporting clinical data is a pillar for international ethical, scientific, and standardized clinical practice. Compliance with this standard provides public assurance being consistent with the principles developed by the Declaration of Helsinki, and that the clinical trial data credibility [12]. Patient records provide continuous information about patients’ medical treatment [13]. They are essential for the patients’ present and future healthcare decision making. Hence, record officers and clerks who generate and process medical data should have sufficient and basic education to spell patient names as well as other related files accurately and correctly [14].

The input data is processed into output information by the information system [15]. High standard healthcare can be ensured by the quality of the data entered the system. This has an important impact on government budget to sustain health service provision [9]. Therefore, accurate and relevant information becomes vital in assisting governments’ decision making regarding service quality [16]. Subsequently, these record systems need to be stored in a manner that maintains integrity; and secures information confidentiality [6].

Information systems include manual and computerized data Technologies. But, the current paper-based record system is inadequate in terms of documentation, disruption, and substantial delay in the healthcare services. Advanced information technologies, on the hand, provide clinicians with real-time information access [17]. Accurate, relevant and structured information is capable of promoting excellent clinical care, measuring efficiency of treatment, and facilitating the interaction of healthcare professionals [9].

According to Clinical Laboratory Standard Institute (CLSI), guidelines should be reviewed at least annually in order to ensure appropriate processes and competence. A quality manager is responsibility for ensuring, controlling, and maintaining evidence for effective operation [18]. Records need to be stored so as to maintain integrity, protect accessibility, and facilitate data retrieval system [8]. Therefore, the implementation of security mechanisms as a highly dynamic procedure must be handled far outside the end-users’ view on domain-specific use cases [19].

### Electronic Health Record (EHR)

Electronic Health Record (EHR) captures, transmits, receives, stores, retrieves, links, and manipulates multimedia in providing health-related services [20]. EHR facilitates the communication of patient information among different professionals [21]. These support tools potentially reduce memory strains of clinicians and improve efficiency and effectiveness in healthcare quality improvement [22]. The accessible patient information has tremendous potential to reduce errors, and support functions. As it is depicted in Table 1 below, these roles or functions include:

**Memory aid:** Reduces the information need to rely on mental memory to complete a task.

**Computational aid:** Reduces the need to mentally compare or analyze information.

**Decision Support aid:** Enhance and integrate information from sources to make decisions.

**Collaboration aid which:** Enhances information communication among providers and patients.

**Table 1:** Expected electronic health record tasks

**Key tasks**	**Electronic health record role**			
	**Memory**	**Computation**	**Decision support**	**Collaboration**
Review Patient History	Display available Patient History & demographics	Provide contextual view of overall patient health	Recommend care based on patient characteristics	Incorporate information from outside sources
Conduct pt Assessment	Prompt for required information	Compute statistics	Action oriented clinical remainders	Coordinate across multiple providers
Determine clinical decision	Relate assessment to patient history	Display trends, reference ranges	Support based on research & recommendations	Staff views or instructions
Develop Treatment Plan	Standard of care, care plans, evidence based guidelines	Apply standards of care based on patient characteristics	Evidence based care adjusted by patent characteristics.	Patient Summary educational tools
Order additional service	Review previous services	Determine appropriate provider	Alignment with insurance requirements	Create referral facility provider communication
Prescribe medications	Medication history allergies, formulary	Dose calculation	Instructions, contraindication, effectiveness	Patient instructions side effects and warnings
Document Visit	Diagnosis and treatment codes	Prompts/automatic population	Insurance guidelines	Patient education, coordination with multiple providers

Source: Armijo *et al*., [22].

EHR holds great promise and success in improving safety, efficiency, timeliness and quality of healthcare with special emphasis is given to interface support tools and secured confidentiality [23].

The quality of records generated and maintained is a reflection of the quality of the healthcare provided. Record management as well as accountability is therefore the cornerstone of good clinical practices. This alerts healthcare providers to prepare and make explicit rationale for decisions making and justify service in the context of evidence-based practices [24].

Computerized documentation may improve or worsen information availability. It could lead to less reliable and less trustworthy documentation than the former paper notes. All responsible groups are expressing their worry about the risk of careless copying and pasting of texts which is less trusted. Moreover, clinicians experience pressure from their stakeholders to document the services provided related to reimbursement. However, there were problems related to disorganized processes of simple insertion laboratory results, vitals, medication lists, and problem lists that could be misleading [25].

### Research findings on record and documentation of patient data

In the United States medical error results in 44,000 to 98,000 unnecessary deaths and 1,000,000 excess injuries per year. Rate of error often increases when inexperienced clinicians introduce new procedures. It had more severe impact associated to extremes of age, complex care, and prolonged hospital stay [26].

Sixty five (3.5%) of 1,934 prescribed agents, Swiss University Hospitals, have committed medical errors. Forty three percent of patient charts showed at least one error. Prescribing errors were found 39 times (37%), transcription errors 56 times (53%), and administration documentation errors 10 times (10%). The handwriting readability was rated as good in 2%, moderate in 42%, bad in 52%, and unreadable in 4%. Higher incidence of documentation error was revealed in the traditional handwritten prescription process. Most errors occurred when prescriptions were transcribed into the patients’ chart. The readability of the handwritten prescriptions was generally bad. Replacing the traditional handwritten documentation process with information technology could potentially improve safety in medication process [27].

Over 14,000 external laboratory results of 128 patients for liver transplant were received from 85 facilities and added to the interfaced EHR at Intermountain HealthCare Center in 2004. It demands regulatory, logistic, economic, and data quality concerns of stakeholders. Coded laboratory data stored in an EHR had several advantages. First, data are accessible from inpatient and remote locations. Secondly, the EHR is permanent and access can be audited. Thirdly, data can be arranged in different views. Finally, electronic information can drive decision support applications [28].

Medical laboratory accreditation schemes assess laboratories meeting accepted standards and providing external validation that ensure the clients accurate, traceable and reproducible services. Well-functioning quality management system, high technical competence, timely and customer-focused services are crucial concerns of accredited laboratories. It demands leadership, time, attention, resources, and continuous commitment to the evaluation and improvement of the processes [29].

In Brigham Women’s Hospital 100,000 patients data were successfully acquired. The dataset included 272,749 coded problems, 442,658 coded medications and 11,801,068 coded laboratory results from the EHR system. There were 1756 unique coded problems, 2128 unique medications and 1341 unique coded laboratory results. The dataset programs were run approximately within 9 minutes to the actual analysis step which was very short [30].

Since March 2006, 29,944 smear microscopy, 31,797 culture and 7,675 drug susceptibility test results have been entered into EHR system. Over 99% of these results have been online accessed by the health centers. This ensured high user satisfaction, heavy use and the expansion of e-Chasqui to additional institutions. EHR provided the service network of institutions and enabled medical care for over 3.1 million people [31].

The study conducted in Sweden showed that the average work time of 50% of the medical staff in western hospitals was spent in searching, registering, and reproducing patient information. They were also spending much money for information processing. Approximately 20% of work time is spent for searching earlier information. The study was also showed more than 10% of laboratory results never reach the responsible ward doctor. Therefore, the implementation of better information system can realize tremendous benefits for all concerned bodies. It decreases the non productive work time of patients by shortening the time on treatment and providing quality healthcare services [32].

Designing and developing hospital information system is an important indicator of quality. Hence, system designing have to consider feasibility, flexibility, robustness, scalability and maintenance which are the basic design principles of system integration [33]. In America, unless otherwise provided by law, all patient records must be retained for at least six years [34]. Widespread adoption of Electronic Medical Record (EMR) system model is safe and eventually could save more than $81 billion annually. This enabled prevention and management of chronic disease and other social benefits. EMR systems could produce savings of $142–$371 billion [35].

A study done in Iran on 300 patient charts showed quality problems in all of them. Interviewed physicians and nurses responded poor hand writing, missing of sheets and incomplete documentation were the major problems of the Paper Based Medical Records (PBMR). Sixty percent of physicians and eighty percent of the nurses believed retrieving of patient information from PBMR was difficult. Ninety percent of the interviewed physicians and most of nurses considered poor hand writing as the main problem of PBMR. More errors related to poor hand writing were committed by physicians than nurses. Nurses believed that most of their working hours were spent on documentation tasks [36].

## Result

E-health is an emerging information and communication technology used to improve the quality of healthcare delivery. Reliable and effective information communication is crucial element in public health practices. The use of appropriate technologies can increase the quality of information and facilitate communication [37]. Therefore, information processing and transmission of knowledge by electronic means is possible through Information Communication Technology (ICT). It enables processing and transmission of information and sharing of knowledge by electronic means [38]. WHO also describes health telematics as a composite term for health-related activities, services and systems like teleconsultation, telediagnosis, remote second opinion, teleradiology, telesurgery, telecare, teleducation and teletraining [38,39].

Online access to patient clinical records from pocket and hand-held or tablet computers will be as useful tool for healthcare. Some of the advantages are better information accessibility, confidentiality, quality improvement and data homogenization. Integrated scientific information system help doctors’ in decision making, minimize the mistakes and to increase the patient safety [40].

## Discussion

Health service provision is a team work. There must be a system to communicate patient information among health professionals and non-professionals involved in different activities of the service delivery processes. Quality management system essentials are common for any kind of work operations [1]. Document and record is one of the 12 QSEs which is important for a smooth running of patient service provision activities [1,19]. Therefore, quality of data is crucial for patient care and monitoring the performance of health service and employees [9]. Quality medical record ensures the extent of stable process of the hospital administration [11]. Clinical record facilitates communication among service providers and hence supports quality of healthcare [6]. Government officials use patient related information for resource allocation, planning, budgeting and other required decisions [16]. It is also an accreditation requirement to have well established documentation and reporting [3,5,18,30].

Quality depends on regulations, standards, guidelines, training and education and accreditation [4]. Health service professionals like nurses and midwives must practice explicit rationale to make and justify decision in the context of legislation, professional standards and guidelines [24].

Record provides evidence based patient care [22], hospital accountability [11], compliance to guideline [18] and support to clinical decision making [22]. EHR holds great success promises and improves the quality and efficiency of health care [22]. Coded laboratory data stored in an EHR are accessible from inpatient and remote locations [28]. Replacing traditional paper based documentation with information technology could potentially improve safety in the medication process [27,36]. Averagely, western world hospital medical staffs spend 50% of their work time in searching, registering and reproducing information. They are spending much money for information processing. Approximately 20% of work time is spent for searching earlier information. In addition, more than 10% of laboratory results never reach the responsible ward doctor [33].

A study in Sweden estimated potential saving because of widespread adoption of EMR systems. An effective EMR implementation and networking could eventually save more than $81 billion annually. The adoption of interoperable EMR systems could produce efficiency and safety savings of $142–$371 billion [37].

ICT is increasingly used to enable healthcare accessibility in remote locations where distance and time is a factor. It increases the quality of healthcare services [38,39]. Healthcare professionals exchange vital information using ICT. Exchange of patient clinical record from pocket and hand held tablet computer through online access is useful and valuable for information delivery. However, internet development could present new threat, risks and challenges [19]. Computerized documentation might both improve and worsen information availability. Therefore, the implementation of security mechanisms in a rather highly dynamic environment alerts a stable, security services through ICT [25].

The delivery of remote healthcare, telemedicine as well the eHealth and newly created Telehealth, that covers almost all aspects of remote Health, is developing almost everywhere. It crosses borders, long distances, cultures, languages, formats, and makes medicine and health care so close to the patients and health professionals as never before. Each national telemedically oriented community chooses its own way to enter and forward telemedicine projects, advancing international knowledge about its applicability, opportunities, possibilities and obstacles and barriers, as well [39].

Real time access of patient clinical record using mobile telephones is very important to make timely decisions. Special design consideration should be given to the processing power and physical constraints of the pocket or hand-held devices [40].

## Conclusion

Health service provision involves multi-professionals. As a team work approach, well established and standardized communication system is mandatory to ensure quality service. Therefore, integrating ICT in healthcare communication system is important for the advancement of quality service. Traditional paper based information communication system has mainly failure costs related to time and quality issues. Employing well designed, secured, user friendly and institutional customizable dynamic information communication mechanisms are important in the competitive, customer centered and highly interactive world. Medical data recording and documentation is a worldwide problem. Many countries have been complaining about incompleteness, suitability and illegibility in recording. As the result patients are dying and/or getting many sufferings due to medical errors.

Access of patient data/information through advanced technology avoids barrier to service provision. The government and health institutions could provide necessary training on data recording, documentation, information processing and communication. Managers should supervise the quality of data, give and/or take moral, ethical, professional and legal responsibilities regarding data completeness, timeliness and correctness.

## Abbreviations

CLSI: Clinical Laboratory Standard Institute; EMR: Electronic Medical Record; ICT: Information Communication Technology; LIS: Laboratory information system; NCCLS: National clinical chemistry laboratory standards; QSE: Quality System Essential; USA: United States of America; PBMR: Paper-based medical records; WHO: World Health Organization.

## Competing interests

The authors declare that they have no competing interests.

## Authors’ contributions

KA; reviewed journals, prepared draft manuscript, finalizing and communication for publication. DM; reviewed journals, drafting the manuscript. MA; Reviewed journals prepared and revised the manuscript. All authors read and approved the final manuscript.
